# Tuberculous Lymphadenitis in Children: A Coincidental Diagnosis—A Case Report

**DOI:** 10.1155/crid/8893555

**Published:** 2025-11-09

**Authors:** Merieme Lferde, Youssef Amal, Hanae Hessissen

**Affiliations:** Department of Pediatric Dentistry, Faculty of Dental Medicine, Mohammed V University, Rabat, Morocco

**Keywords:** case report, cervical tuberculous lymphadenitis, cervicofacial swellings, extrapulmonary tuberculosis, pediatrics, tuberculosis

## Abstract

**Background:**

The discovery of a cervicofacial swelling is a common concern among the pediatric population with a broad range of potential diagnoses. While infectious origins related to dental infection are frequent, it is crucial not to overlook other systemic causes. Among these causes, cervical tuberculous lymphadenitis (CTL) stands out as one of the most common forms of extrapulmonary tuberculosis. This condition poses a significant diagnostic challenge in children, as it is often asymptomatic and can lead to a misdiagnosis.

**Case Presentation:**

The aim of this paper is to highlight, through a case report, the diagnostic approach to a lower mandibular swelling in a 10-year-old boy initially diagnosed as cellulitis of dental origin. However, further investigation revealed CTL, confirmed by lymph node biopsy, leading to a 6-month regimen of four drugs for treatment. This uncommon presentation emphasizes the importance of thorough investigation, as the overlap in symptoms with dental infection could potentially lead to misdiagnosis. Dentists can play a crucial role in detecting tuberculosis in cases of cervicofacial swelling. By doing so, they can ensure early diagnosis and promptly refer the patient for appropriate management.

**Conclusion:**

The case report underscores the importance of considering tuberculosis in the differential diagnoses of cervicofacial swellings in children, emphasizing comprehensive clinical, radiological, and histopathological assessment for accurate diagnosis and effective treatment.

## 1. Introduction

Cervicofacial swellings are a frequent reason for consultation in pediatric dentistry, causing considerable anxiety for parents. Their etiologies are numerous and their diagnosis is most frequently correlated with cellulitis of dental origin; nevertheless, a neoplastic or systemic origin should not be omitted.

Lymphadenopathy refers to changes in the size, number, or consistency of lymph nodes due to infiltration by inflammatory or malignant cells. Cervical lymphadenopathy involves enlargement of cervical nodes (> 1 cm) and is typically a temporary response to infection but may occasionally indicate underlying systemic conditions like autoimmune diseases or malignancies. Lymphadenitis distinctively refers to lymphadenopathies having an inflammatory origin [1].

Cervical tuberculous lymphadenitis (CTL) is the most common cause of persistent cervical lymphadenitis in the pediatric group and developing countries. It typically presents as a slowly enlarging, painless swelling of lymph nodes over weeks to months. Early-stage nodes are firm, mobile, and nontender but may later become matted with inflamed skin. Advanced stages involve softening, abscess formation, and sinus tract development. Systemic symptoms like fever, weight loss, fatigue, and night sweats are seen in more extensive disease. In severe cases, enlarged nodes may compress or invade nearby structures, complicating the clinical course [2].

Delayed or misdiagnosis of CTL can lead to unnecessary treatments, disease progression, and an increased risk of transmission. Despite its clinical importance, CTL is often underrecognized because of its nonspecific presentation and overlap with other cervical pathologies such as bacterial lymphadenitis, neoplasms, or systemic diseases [3].

This paper proposes to illustrate, through a clinical case, the diagnostic approach of a lower mandibular swelling in a 10-year-old patient affected by CTL which mimicked dental cellulitis. This highlights the importance of a comprehensive diagnostic approach, as the overlap in symptoms with dental infection could potentially lead to misdiagnosis.

## 2. Case Presentation

### 2.1. Patient Information

A 10-year-old male was referred to the Department of Pediatric Dentistry, complaining of a swelling on the left side of the lower jaw for the last 2 months. The anamnesis revealed that the patient was in good health, with no history of fever, cough, or weight loss. Additionally, the patient had self-administered amoxicillin a month ago (1 g per day for 5 days).

### 2.2. Clinical Findings

**Figure 1:**
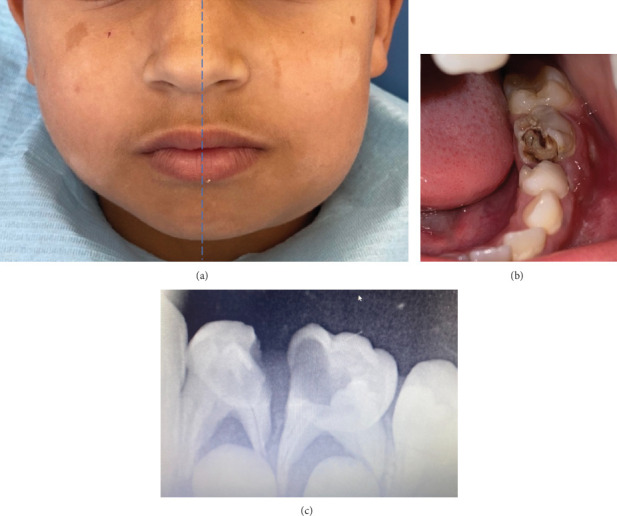
(a) On examination, the face appeared asymmetrical with a swelling in the left cheek. Overlying skin was of normal color. Mouth opening was 25 mm. (b) Endobuccal view showing Teeth 74 and 75 with twin cavities and a buccal suppurated collection. (c) Retroalveolar radiography of Teeth 74 and 75 showing the pulpoperiodontal implications.

On clinical examination, a poorly delimited, nonerythematous, depressible in its center, and painless lower left angulomandibular swelling was noted with multiple lymphadenopathy on both sides (Figure 1a). Salivary flow of all glands was normal.

Intraoral examination revealed poor oral hygiene and the presence of two decayed teeth (74 and 75) accompanied by a painful suppurating swelling of the buccal fold (Figure 1b). A retroalveolar radiography confirmed the diagnosis of pulpoperiodontal pathologies for both teeth (Figure 1c).

### 2.3. Diagnostic Assessment

Based on these findings, an initial diagnosis of cellulitis of dental origin was retained.

### 2.4. Therapeutic Intervention

The etiologic treatment involved the extraction of the two affected teeth.

### 2.5. Follow-Up and Outcomes

**Figure 2:**
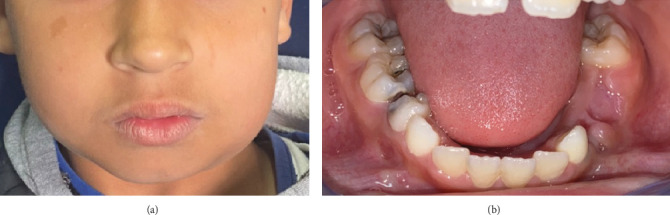
(a) At the control visit, the swelling regressed and became more limited to the submandibular region. (b) Three weeks after the extraction of Teeth 74 and 75, the buccal collection completely disappeared.

At the time of the follow-up sessions, the buccal suppurative collection had completely regressed, unlike the cervicofacial swelling. Although it had diminished and become more confined to the submandibular area, it has not disappeared (Figure 2a,b).

**Figure 3:**
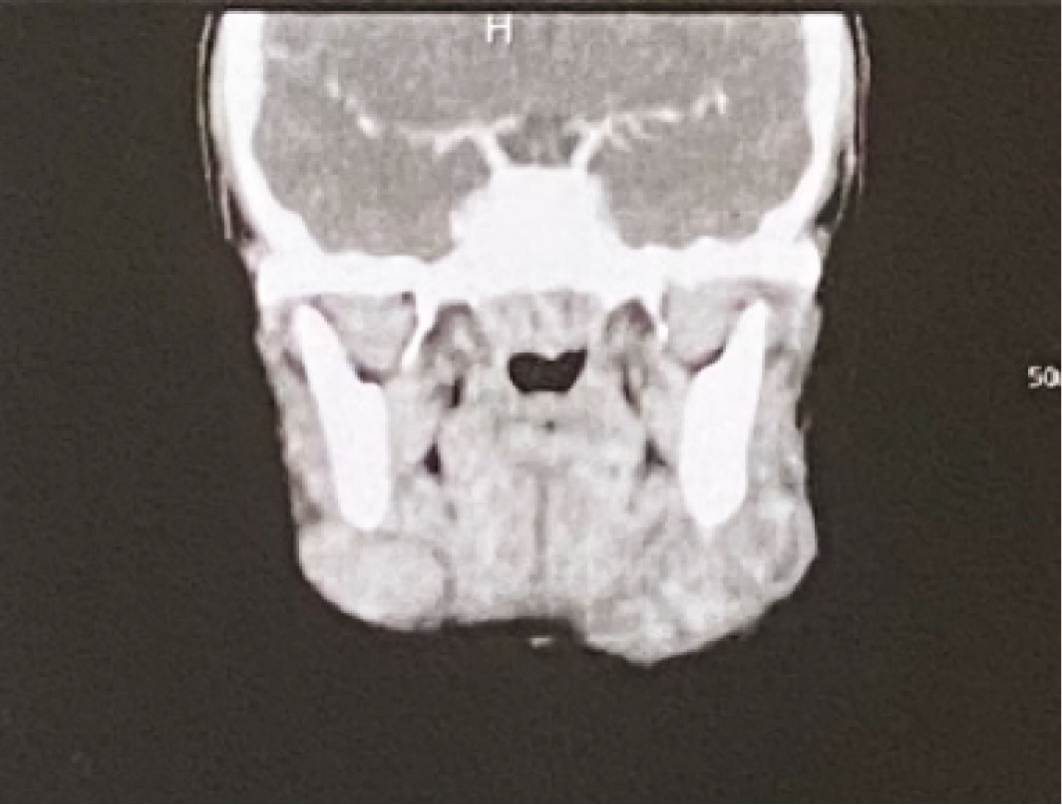
Computed tomography scan of the neck showing enlarged bilateral lymph nodes. On the patient's left, the largest lymph node has a central hypodensity consistent with necrosis.

Hence, a clinical differential diagnosis of submandibular lymphadenitis or lymph node tumor was made, justifying a medical ultrasound and a complete blood count that were requested from the patient. The ultrasound revealed findings consistent with benign lymphadenopathy (homogeneous echo structure with a clean and regular contour), and the blood count did not show any abnormalities. This allowed us to rule out the cancerous origin of the lesions, particularly related to a lymphoma. In addition, an exploratory cervicofacial scan revealed that the lymphadenopathy could be compatible with a tuberculosis (TB) origin due to the detection of caseous necrosis (Figure 3). The intradermal tuberculin test was positive, which was also in favor of a *Mycobacterium tuberculosis* infection. A lymph node biopsy subsequently confirmed the final diagnosis of CTL.

The extension of the disease had been assessed by a chest radiography, which was negative. The medical treatment consisted of a 6-month regimen of four drugs: isoniazid, rifampicin, ethambutol, and pyrazinamide. CTL responds well to antimycobacterial regimens and has a good prognosis. The patient has not reported to our department for further follow-up.

### 2.6. Informed Consent

Written informed consent for the case to be published was obtained from the patient and her mother for publication of this case report, including accompanying images.

## 3. Discussion

Until the coronavirus (COVID-19) pandemic, TB was the leading cause of death from a single infectious agent, ranking above HIV/AIDS [4]. About a quarter of the world's population is infected with *M. tuberculosis*, equivalent to about 2 billion people. The probability of developing TB disease is much higher among people living with HIV and among people affected by risk factors such as undernutrition, diabetes, smoking, and alcohol consumption [5].

Transmission of TB occurs via aerosolized droplets, which seed organisms into the lungs that persist viably within macrophage after ingestion. Often called “primary TB infection,” this site is asymptomatic [6]. A reactivation of this primary disease is possible, either by reinfection or change in the individual's immune status. TB typically affects the lungs (pulmonary TB) but can also affect in some cases other sites (extrapulmonary TB), when lymphatic or hematogenous spread occurs [7]. The extrapulmonary form is dominated by cervical lymphadenitis involvement [8].

CTL, also named “scrofula” or “the king's evil,” is a major challenge because its nonspecific clinical findings may overlap with other diseases [9]. In our case, it was masked by cellulitis of dental origin. The presentation of a case (CTL) mimicking dental cellulitis is rare and provides a valuable case study. This highlights the importance of a thorough clinical and complementary examination as well as a rigorous follow-up for complex cases.

Given the limited diagnostic value of clinical examination in suspected submandibular TB, it is crucial to consider a broad spectrum of differential diagnoses. In our case, submandibular salivary gland pathology was ruled out by palpation and normal salivary flow. Hematological evaluation did not reveal abnormalities suggestive of lymphatic neoplasms, and ultrasound findings showed benign lymphadenopathy with a homogeneous echo structure and regular contour, ruling out a malignant process. An exploratory cervicofacial scan further raised suspicion of TB due to the detection of caseous necrosis. Importantly, differential diagnoses such as granulomatous diseases (e.g., sarcoidosis), neoplastic causes (e.g., lymphoma and metastatic carcinoma), and other infectious conditions (bacterial or viral lymphadenitis) must also be systematically considered and excluded.

Ultimately, confirmation of CTL was achieved through lymph node biopsy. This comprehensive diagnostic approach highlights the importance of integrating clinical, imaging, and histopathological findings to accurately diagnose TB, especially in cases with atypical presentations.

Histopathological analysis and tuberculin skin testing remain valuable tools in the diagnostic process. However, newer rapid diagnostic techniques such as molecular assays, particularly GeneXpert *M. tuberculosis* Branching MTB/RIF and PCR-based methods, have demonstrated higher sensitivity and specificity and allow simultaneous detection of rifampicin resistance. These tools are increasingly accessible in clinical practice and can facilitate earlier and more accurate diagnosis, particularly in extrapulmonary forms where conventional diagnostic modalities may be limited [10].

Epidemiologically, these lymphadenopathies are reported to be predominantly female [11], which does not match our case. Many patients will present one or two large, indurated, fixed nodes with a suspicion of fluctuation due to necrosis, as in our case. Others will present several smaller, firm nodes that are freely removable.

Cervical lymph node excision will confirm diagnosis and resolve the involved node, but the other cervical lymph nodes may be infected in an earlier stage of the disease or may harbor mycobacteria [3]. Therefore, patients require the same systemic drug regimens as for pulmonary TB. In addition, a chest radiograph and a thorough survey for other areas of TB are warranted [12].

Spontaneous healing, although possible, is rare and takes years to achieve. It responds well to antimycobacterial regimens for TB and has a good prognosis. Nonetheless, outcomes can vary depending on the disease severity, the immune status of the patient (notably HIV coinfection), delays in diagnosis, and resource limitations in certain settings. These factors may influence treatment success and the risk of recurrence [13].

Moreover, given that *M. tuberculosis* is transmitted via aerosols, infection control measures are essential, especially in dental settings where aerosol-generating procedures are frequent. Dentists and oral health professionals should adhere strictly to infection prevention protocols, including proper sterilization of instruments, use of personal protective equipment (PPE), meticulous hand hygiene, N95 respirators when indicated, rubber dams to reduce aerosol spread, and thorough disinfection of surfaces after procedures. These measures are critical to prevent nosocomial infections and occupational exposure [14].

From a management standpoint, treatment adherence is the cornerstone of successful outcomes. Irregular or incomplete therapy is the main cause of treatment failure and drug-resistant strains. Therefore, patient education, psychosocial support, and close follow-up are essential throughout the prolonged treatment period (often ≥ 6 months) [15]. In resource-limited settings, directly observed therapy (DOT) remains an effective strategy to ensure adherence and prevent multidrug resistance [16].

Finally, clinicians must monitor for adverse drug reactions, including hepatotoxicity, optic neuritis (ethambutol), and peripheral neuropathy (isoniazid). Regular clinical evaluations and biochemical monitoring, when possible, allow early detection of toxicity and timely management. This vigilance is particularly crucial in immunocompromised patients, such as those with HIV, who face increased risks of drug interactions, immune reconstitution inflammatory syndrome, and poorer outcomes [17]. A multidisciplinary approach integrating infectious disease specialists, dentists, and primary care providers optimizes diagnosis, monitoring, and long-term prognosis.

## 4. Conclusion

To sum up, cervicofacial swellings can stem from various etiologies that may coexist in the same patient. Differential diagnosis relies on a thorough clinical and complementary examination, along with rigorous follow-up. This case underscores the importance of detecting unusual pathologies of the oral cavity, facilitating early diagnosis and prompt referral for appropriate management. The uncommon presentation highlights the necessity of thorough investigation, as symptoms overlapping with dental infection could potentially lead to misdiagnosis. Dentists play a crucial role in detecting TB in cases of cervicofacial swelling, ensuring early diagnosis and timely referral for proper management. This proactive approach is indispensable for preventing treatment delays and potential complications associated with TB, contributing significantly to enhancing patient outcomes and overall public health.

## 5. Patient and Family Perspective

From the patient's and family's perspective, the experience was initially stressful. The child presented with a swelling initially suspected to be of dental origin, leading to multiple consultations and investigations before TB was confirmed.

Once the diagnosis of CTL was established, clear explanations from the medical team and reassurance about the disease's curability helped ease their concerns. The family expressed appreciation for the multidisciplinary approach and the supportive communication maintained throughout care. Adherence to therapy was facilitated by parental involvement, understanding of the treatment plan, and regular monitoring visits that reinforced their confidence in the care process.

## 6. Recommendations and Take-Home Message

• Maintain a high index of suspicion for extrapulmonary TB in children presenting with persistent cervical swellings, especially when initial management for common causes (e.g., odontogenic or bacterial infections) fails.
• Early referral and multidisciplinary evaluation (dentist, pediatrician, infectious disease specialist, and radiologist) are crucial to avoid diagnostic delays.
• Use of molecular diagnostic tools such as GeneXpert MTB/RIF improves early detection and guides therapy, particularly in resource-limited settings.
• Infection control precautions must be rigorously observed in dental and clinical environments to prevent aerosol transmission of *M. tuberculosis*.
• Parental education and psychosocial support play a key role in ensuring treatment adherence and minimizing anxiety throughout the prolonged therapeutic course.
• This case underscores the need for heightened clinical awareness among dental professionals, who may be the first to encounter such presentations, and the importance of considering TB in the differential diagnosis of persistent cervicofacial swellings in children.

## Data Availability

All data generated or analyzed during this study are included in this published article. Further inquiries can be directed to the corresponding author.
